# Reliability of a novel approach for reference-based cell type estimation in human placental DNA methylation studies

**DOI:** 10.1007/s00018-021-04091-3

**Published:** 2022-02-03

**Authors:** Linda Dieckmann, Cristiana Cruceanu, Marius Lahti-Pulkkinen, Jari Lahti, Tuomas Kvist, Hannele Laivuori, Sara Sammallahti, Pia M. Villa, Sanna Suomalainen-König, Rebecca C. Rancourt, Andreas Plagemann, Wolfgang Henrich, Johan G. Eriksson, Eero Kajantie, Sonja Entringer, Thorsten Braun, Katri Räikkönen, Elisabeth B. Binder, Darina Czamara

**Affiliations:** 1 Department of Translational Psychiatry, Max Planck Institute of Psychiatry, Munich, Germany; 2 International Max Planck Research School for Translational Psychiatry, Munich, Germany; 3 Department of Psychology and Logopedics, Faculty of Medicine, University of Helsinki, Helsinki, Finland; 4 Finnish Institute for Health and Welfare, Helsinki, Finland; 5 Centre for Cardiovascular Science, Queen’s Medical Research Institute, University of Edinburgh, Edinburgh, UK; 6 Institute for Molecular Medicine Finland, HiLIFE, University of Helsinki, Human Genetics, Helsinki, Finland; 7 Medical and Clinical Genetics, University of Helsinki and Helsinki University Hospital, Helsinki, Finland; 8 Department of Obstetrics and Gynecology, Faculty of Medicine and Health Technology, Center for Child, Adolescent and Maternal Health, Tampere University Hospital and University of Tampere, Tampere, Finland; 9 Children’s Hospital, Helsinki University Hospital and University of Helsinki, Helsinki, Finland; 10 Department of Child and Adolescent Psychiatry, Erasmus MC, Sophia Children’s Hospital, Rotterdam, The Netherlands; 11 Department of Obstetrics and Gynecology, Helsinki University Central Hospital, Helsinki, Finland; 12 Hyvinkää Hospital, Helsinki and Uusimaa Hospital District, Hyvinkää, Finland; 13 Department of Experimental Obstetrics, Charité-Universitätsmedizin Berlin, Corporate Member of Freie Universität Berlin, Humboldt-Universität Zu Berlin, and Berlin Institute of Health (BIH), Berlin, Germany; 14 Department of Obstetrics, Charité-Universitätsmedizin Berlin, Corporate Member of Freie Universität Berlin, Humboldt-Universität Zu Berlin, and Berlin Institute of Health (BIH), Berlin, Germany; 15 Department of General Practice, University of Helsinki, Helsinki, Finland; 16 Folkhälsan Research Center, Helsinki, Finland; 17 Department of Obstetrics and Gynaecology and Human Potential Translational Research Programme, Yong Loo Lin School of Medicine, National University of Singapore, Singapore, Singapore; 18 Singapore Institute for Clinical Sciences, Agency for Science, Technology and Research (A*STAR), Singapore, Singapore; 19 Faculty of Medicine, PEDEGO Research Unit, MRC Oulu, Oulu University Hospital and University of Oulu, Oulu, Finland; 20 Department of Clinical and Molecular Medicine, Norwegian University of Science and Technology, Trondheim, Norway; 21 Institute of Medical Psychology, Charité-Universitätsmedizin Berlin, Corporate Member of Freie Universität Berlin, Humboldt-Universität Zu Berlin, and Berlin Institute of Health (BIH), Berlin, Germany; 22 Department of Pediatrics, Development, Health, and Disease Research Program, University of California, Irvine, CA USA; 23 Department of Psychiatry and Behavioral Sciences, Emory University School of Medicine, Atlanta, GA USA

**Keywords:** Cell type estimation, DNA methylation, Human placenta, Chorionic villi, Reference-based deconvolution, Reference-free deconvolution

## Abstract

The placenta is a central organ during early development, influencing trajectories of health and disease. DNA methylation (DNAm) studies of human placenta improve our understanding of how its function relates to disease risk. However, DNAm studies can be biased by cell type heterogeneity, so it is essential to control for this in order to reduce confounding and increase precision. Computational cell type deconvolution approaches have proven to be very useful for this purpose. For human placenta, however, an assessment of the performance of these estimation methods is still lacking. Here, we examine the performance of a newly available reference-based cell type estimation approach and compare it to an often-used reference-free cell type estimation approach, namely RefFreeEWAS, in placental genome-wide DNAm samples taken at birth and from chorionic villus biopsies early in pregnancy using three independent studies comprising over 1000 samples. We found both reference-free and reference-based estimated cell type proportions to have predictive value for DNAm, however, reference-based cell type estimation outperformed reference-free estimation for the majority of data sets. Reference-based cell type estimations mirror previous histological knowledge on changes in cell type proportions through gestation. Further, CpGs whose variation in DNAm was largely explained by reference-based estimated cell type proportions were in the proximity of genes that are highly tissue-specific for placenta. This was not the case for reference-free estimated cell type proportions. We provide a list of these CpGs as a resource to help researchers to interpret results of existing studies and improve future DNAm studies of human placenta.

**Supplementary Information:**

The online version contains supplementary material available at 10.1007/s00018-021-04091-3.

## Introduction

Since the *Developmental Origins of Health and Disease* (DOHaD) hypothesis was proposed, converging evidence supports the high importance of intrauterine conditions for development, as well as for health and disease outcomes later in life [1–3]. The placenta is a complex organ with a central role in fetal development and regulation of the intrauterine environment throughout pregnancy [4–6]. Thus, a better understanding of the placenta’s critical role for early development and its molecular landscape is key to disentangling some of the mechanisms driving DOHaD-related developmental aspects [7]. Epigenetic processes are essential for placental development and function, and correspondingly healthy fetal development [8, 9]. Consequently, human studies of the placental epigenome are valuable and can help to increase our knowledge about trajectories of health and disease originating in early life.

DNA methylation (DNAm) is one of the most commonly studied epigenetic marks and it is known to be highly tissue- and cell-type-specific. Accordingly, it is important to distinguish direct (true) associations between the exposure of interest and DNAm from associations mediated trough or otherwise caused by placental cell type distributions [10, 11].

To this end, cell type deconvolution algorithms have been developed to retrieve information about cell type composition from DNAm data. They can be mainly categorized into reference-based and reference-free methods [10]. Reference-based cell type deconvolution algorithms rely on biologically defined 5′-C-phosphate-G-3′ (CpG) sites that are uniquely methylated in purified cell types and were identified in a reference sample. For reference-free deconvolution, no a-priori knowledge about differential methylation from purified cell types is necessary, but cell types are predicted directly from DNAm using a computational approach [12]. The first reference-based method to infer changes in the distribution of white blood cells using DNAm signatures was proposed in 2012 by Houseman et al. [13], and pioneering algorithms for reference-free cell type deconvolution were published in 2014 [14, 15]. While reference-free methods are useful when no reference is available, reference-based methods are preferred if a reference is available and there is no evidence for other confounders [10, 16]. To date, the effectiveness of reference-free cell type deconvolution for placenta has not been assessed, and only recently a reference profile for placenta was published [17]. The establishment and validation of this reference in 28 samples constitutes important progress and now allows a reference-based cell type estimation in placenta.

However, an assessment of the performance of this reference-based versus reference-free cell type estimation in placenta with larger study samples is crucial for informing future research. In the current study, we demonstrate the impact of reference-based versus reference-free estimated cell types on DNAm in placental tissue and compare their informativeness. Further, we provide an overview of estimated cell types in placental samples from three independent studies, taken at birth (*n* = 470, *n* = 139, *n* = 137) and, in the largest of these three studies, also during the first trimester (*n* = 264). Our study contributes to a more detailed understanding of human placental characteristics regarding the relatedness of DNAm and cell type composition and underscores the importance of considering cell types in future DNAm studies using placental tissue.

## Materials and methods

### Study populations

Placental tissue samples were collected from the InTraUterine sampling in early pregnancy (ITU) study, the Prediction and Prevention of Preeclampsia and Intrauterine Growth Restriction (PREDO) study [18], and the Betamethasone (BET) study [19].

ITU and PREDO are Finnish cohort studies consisting of women and their children who were followed throughout pregnancy and beyond. In ITU, women were recruited through the national voluntary prenatal screening program for trisomy 21. If this screening indicated an increased risk of fetal chromosomal abnormalities based on routine serum, ultrasound screening, age and patient history, women were offered further testing including chorionic villus sampling (CVS) at the Helsinki and Uusimaa Hospital District Feto-maternal Medical Center (FMC). During this visit, women were informed about the ITU study. If the chromosomal test indicated no fetal chromosomal abnormality, those who had expressed interest in participating were contacted for final recruitment. Another set of women were informed about ITU when attending the routine screening at maternity clinics. If interest in participating was expressed, they were contacted for final recruitment into the ITU study. In PREDO, the recruitment took place when women attended their first routine ultrasound screening. Some of the women were recruited based on having clinical risk factors for preeclampsia and intrauterine growth restriction, others were recruited independently of these factors [18]. The aim of the BET study was to investigate the effect of antenatal betamethasone on the transplacental cortisol barrier and fetal growth [19]. Pregnant women with preterm labor and cervical shortening were treated with a single course of antenatal BET (Celestan^®^, MSD GmbH, Haar, Germany) for fetal maturation between 23 + 5 and 34 + 0 weeks of gestation and were recruited prospectively before birth. A gestational-age-matched control group consisted of pregnant women who received no antenatal BET.

### Placental tissue samples

In the ITU study, first-trimester placental biopsies were obtained from leftover CVS, following indications of elevated risk for chromosomal abnormalities between 10 and 15 weeks of gestation. Placenta samples were also collected at birth, whereby midwives/trained staff took nine-site biopsies (within maximum 120 min after delivery) from the fetal side of the placenta, at 2–3 cm from umbilical cord insertion. In the PREDO study, placenta nine-site biopsies (within maximum 90 min after delivery) were taken from the decidual side of the placenta. In the BET study, full-thickness placental biopsies were taken by a uniform random sampling protocol [20, 21] from both peripheral and central areas. All samples were stored at − 80 °C.

Throughout the manuscript, we refer to all placental samples collected at birth as ‘term placenta’, and to all placental CVS samples collected during early pregnancy as ‘CVS’.

### DNA methylation (DNAm)

From the collected samples, DNA was extracted according to standard procedures and DNAm was assessed using the Illumina Infinium MethylationEPIC array (Illumina, San Diego, USA). In total, DNA methylation levels were assessed in 1055 samples: *n* = 277 CVS samples (ITU), and *n* = 500 placental samples (ITU), *n* = 140 placental samples (PREDO), and *n* = 138 placental samples (BET) taken at birth. All DNAm data were pre-processed in the same way, using an adapted pipeline from Maksímovíc et al. [22] and the R package *minfi* [23]. Beta values were normalized using stratified quantile normalization [24], followed by BMIQ [25]. Batch-effects were removed using *ComBat* [26].

The final data sets comprised 264 CVS samples from ITU (*n* = 716,331 probes) and 486 placental samples (*n* = 665,190 probes) from ITU, 139 placenta samples (*n* = 755,154 probes) from PREDO and 137 placenta samples (*n* = 708,222 probes) from the BET study. Of these, 652,341 probes overlapped across all four data sets.

### Gestational age, child sex and ethnicity variables

Gestational age (GA) at sampling was based on fetal ultrasound. Child sex was extracted from the Finnish Medical Birth Register (MBR) in ITU and PREDO and obtained from postnatal assessment in the BET study. To retrieve information about genetic background, we performed multi-dimensional scaling (MDS) analysis on the identity-by-state (IBS) matrix of quality-controlled genotypes [27]. We used the first two components for ITU and PREDO and the first four components for the BET study, as it was ethnically more heterogenous. In the following, we refer to these MDS components as ‘PC 1/2/3/4 ethnicity’, respectively. This information was available for *n* = 200 individuals with CVS tissue in ITU, and *n* = 439 individuals with term placental tissue in ITU, in *n* = 118 individuals with term placental tissue in PREDO and *n* = 136 individuals with term placental tissue in BET. Genotyping was performed on Illumina Infinium Global Screening arrays for BET and ITU and on Illumina Human Omni Express Arrays for PREDO. DNA for genotyping was extracted from cord blood in ITU and PREDO, if available, otherwise placental tissue was used in ITU. DNA was extracted from placental tissue in the BET study. Further details about genotypic assessment and quality control in the ITU and PREDO cohorts, as well as in the BET study, have been published elsewhere [28, 29].

An overview of study sample characteristics is given in Table 1.

**Table 1:** Study sample characteristics [*Mean *(*SD*) or *N* (%) for each variable]

	ITU		PREDO	BET
	CVS	Placenta	Placenta	Placenta
Sample size	264	470	139	137
Phenotypes				
Gestational age	12.79 (0.82)	39.99 (1.55)	39.89 (1.43)	38.16 (1.95)
Child sex (male)	140 (53%)	238 (51%)	67 (48%)	70 (51%)
Reference-based cell types				
Trophoblasts	0.26 (0.06)	0.01 (0.03)	0.04 (0.05)	0.13 (0.06)
Stromal	0.17 (0.06)	0.01 (0.02)	0.04 (0.03)	0.11 (0.02)
Hofbauer	0.00 (0.01)	0.00 (0.01)	0.00 (0.00)	0.00 (0.00)
Endothelial	0.00 (0.01)	0.01 (0.02)	0.08 (0.03)	0.11 (0.02)
nRBC	0.00 (0.01)	0.04 (0.03)	0.00 (0.01)	0.00 (0.00)
Syncytiotrophoblasts	0.57 (0.04)	0.93 (0.06)	0.83 (0.08)	0.66 (0.08)
Reference-free cell types				
C1	0.26 (0.14)	0.11 (0.09)	0.43 (0.19)	0.35 (0.2)
C2	0.30 (0.15)	0.07 (0.07)	0.51 (0.20)	0.46 (0.2)
C3	0.14 (0.07)	0.23 (0.13)	–	0.14 (0.1)
C4	0.10 (0.07)	0.13 (0.09)	–	–
C5	0.14 (0.10)	0.13 (0.09)	–	–
C6	–	0.11 (0.08)	–	–
C7	–	0.09 (0.07)	–	–
C8	–	0.08 (0.07)	–	–

### Cell type composition estimation

Reference-based cell type composition into six cell types (nucleated red blood cells, trophoblasts, syncytiotrophoblasts, stromal, Hofbauer, endothelial) was estimated using a reference recently published by Yuan et al. [17] and implemented within the R package *planet*, by applying the robust partial correlation algorithm [30].

The result of this cell type estimation is the amount of the respective cell types in every person, while all estimated cell types add up to 100%.

Reference-free cell types were estimated following the protocol suggested in the R package *RefFreeEWAS* [31], which led to five estimated ‘cell types’ in CVS (ITU), and eight estimated ‘cell types’ (ITU), two estimated ‘cell types’ (PREDO) and three estimated ‘cell types’ (BET) in term placenta. We refer to cell types here, although the output of this procedure does not give explicit cell types, but latent quantities and their respective proportion for every person.

### Statistical analyses

All statistical analyses were performed in R, version 4.0.5/4.1.1 [32].

#### Filtering of invariable probes in DNAm

To assess the influence of cell types on DNAm, we first filtered for variable CpGs by excluding placenta-specific non-variable CpGs. We applied a procedure described by Edgar et al. [33] to the overlapping CpGs (*n* = 652,341) of all four placental methylation data sets from the EPIC array, to identify sites with < 5% range between 10 and 90th percentile in DNAm beta values using our data sets. This resulted in 120,548 CpGs (listed in Supplementary Table S1) that we identified as non-variable for placental EPIC methylation data and excluded from further analyses. Identifying these CpGs is useful to reduce dimensionality, and becomes especially relevant for future studies, e.g., epigenome-wide association studies (EWAS), aiming to use our resources. Furthermore, the 1050 CpGs used to predict cell type composition in the model by Yuan et al. [17] were excluded from the following analyses to prevent circular conclusions.

#### Capturing DNAm variance through principal components and filtering of individuals

To capture the major variance in DNAm, we performed singular value decomposition on methylation beta values, and extracted the first principal component (PC1) explaining most of the variance for every data set (Supplementary Fig. S1). For term placenta from ITU we identified *n* = 16 outliers representing values greater than three times inter-quartile-range in PC1 (see Supplementary Fig. S2a). The same samples showed lower sample-sample correlations in DNAm beta values with the other placenta samples (Supplementary Fig. S2b) and presented different cell type proportions (Supplementary Fig. S2c). Thus, we excluded these samples from the ITU placenta data set, resulting in *n* = 470 term placenta samples from the ITU cohort. We calculated the principal components (PC) without these outliers in the ITU term placenta data set. For CVS from ITU and term placenta data sets from PREDO and BET no such outliers were identified.

#### Correlation of reference-free estimated cell types with reference-based estimated cell types and phenotypes

Spearman's rank correlations were calculated both between reference-free and reference-based estimated cell types and between reference-free estimated cell types and phenotypes (GA, child sex, ethnicity PCs and additionally fetal chromosomal testing and BET administration status in the ITU and BET placenta, respectively) in every tissue. Adjustment for multiple testing was done using Bonferroni correction.

#### Models to predict DNAm by cell type proportions (reference-based versus reference-free)

To compare the impact of reference-based versus reference-free estimated cell types on the main variance in DNAm, PC1 of DNAm beta values was regressed linearly on different predictors in six models for every data set:

1. PC methylation ~ 1
2. PC methylation ~ GA at sampling + child sex + PCs ethnicity
3. PC methylation ~ reference-based estimated cell types
4. PC methylation ~ reference-based estimated cell types + GA at sampling + child sex + PCs ethnicity
5. PC methylation ~ reference-free estimated cell types
6. PC methylation ~ reference-free estimated cell types + GA at sampling + child sex + PCs ethnicity

Using cross-validation with 10 folds, 500 repeats and *RMSE* as loss function, implemented in the R package *xvalglms* [34], enabled us to evaluate which model best explains variability in placental DNAm. This is defined by the number of times a particular model wins in the repeated cross-validation procedure, i.e., the number of times that the model has a smaller prediction error (*RMSE*, in our case) than all other models considered. *RMSE* is on the same scale as the outcome variable and the partitions of data were the same for all models. As *RMSE* is not comparable between the data sets, we additionally report the adjusted *R*^2^ values of the winning models.

For the BET data set, we observed outliers in *RMSE* in some of the repeats (see Supplementary Fig. S3a). After further exploration it became evident that these were driven by five samples, which were different in Hofbauer and nRBC cell type proportions, i.e., all samples apart from these five had no estimated proportions of Hofbauer and nRBC cells (see Supplementary Fig. S3b). We also tested if outliers in any of the other estimated cell types (see Supplementary Fig. S3c) changed the behavior of the model, but this was not the case. Furthermore, outliers were present in all data sets and are not suspicious per se in samples from heterogenous tissue like placenta. Thus, we only excluded the five samples presenting very different in estimated Hofbauer and nRBC cells in the BET data set from this analysis.

We further tested how much of DNAm variability in all single CpGs could be explained by either reference-based or reference-free estimated cell types. Linear models were fitted for every CpG by predicting DNAm (beta values) with either reference-based or reference-free cell types. For every CpG, the adjusted *R*^2^ was extracted (see Supplementary Fig. S4 for a histogram of *R*^2^ values). Afterwards, CpGs with adjusted *R*^2^ > 0.30 in all four data sets were extracted and considered as CpGs at which variability of DNAm (beta values) was relatively strongly influenced by cell type proportions. We decided to use this criterion based on an evaluation of the histograms (Fig. S4) and as the mean adjusted R^2^ values of the 90% quantile of all data sets was *R*^2^_Adjusted_ = 0.30, and our aim to only extract the most informative CpGs, i.e., to be rather strict in this selection. For the following enrichment analyses, the genes (20,038) mapping to all CpGs (534,510) overlapping between the data sets were used as background.

#### Enrichment analyses

All CpGs were mapped to the closest gene using the R package *bumphunter* functions *annotateTranscripts* and *matchGenes* [35]. Afterwards, the genes corresponding to the extracted CpGs were used as input for the *TissueEnrich* package [36], while the genes corresponding to all CpGs overlapping between the data sets (without any filtering for *R*^2^) were considered as background genes (*n* = 20,038). The same input and background genes were further used for the *PlacentaCellEnrich* Tool [37]. Human placental single-cell RNA-Sequencing data [38] were used to retrieve enrichments for placenta cell-specific expression patterns. For both enrichment analyses we used an adjusted *p* value of 0.01 as threshold for enrichment, as recommended by the authors of the *PlacentaCellEnrich* Tool [37].

#### Cell type composition analyses

Differences in reference-based cell type proportions between the three term placenta data sets were analyzed using nonparametric global multivariate analysis of variance [39] implemented in the R package *npmv* [40]. To test for significant differences between the study groups, we applied the global test using the *R* function *nonpartest* with default settings, which provides *F*-distribution approximations, performs multivariate permutation and calculates nonparametric relative effects. The global test was supplemented with a more detailed comparison (*R* function *ssnonpartest*) of study groups and cell types using the *F* approximation of Wilks’ lambda, to identify which variables/factor levels contribute to the significant differences, while controlling for the familywise error rate (*α* = 0.01).

Differences in reference-based cell type proportions between CVS and term placenta from the same individuals (*n* = 85, ITU) were calculated using paired Wilcoxon signed-rank tests. All *p* values were corrected for multiple testing (*n* = 6 cell types) using Bonferroni correction and compared to *α* = 0.01.

Spearman correlations and Wilcoxon signed-rank tests were performed to test for relationships between reference-based cell type proportions and GA and child sex (for every cell type separately and corrected for multiple testing among the *n* = 6 cell types using Bonferroni correction and *α* = 0.01).

## Results

### Reference-free estimated cell types do not map to reference-based estimated cell types and are correlated with child sex

**Fig. 1:**
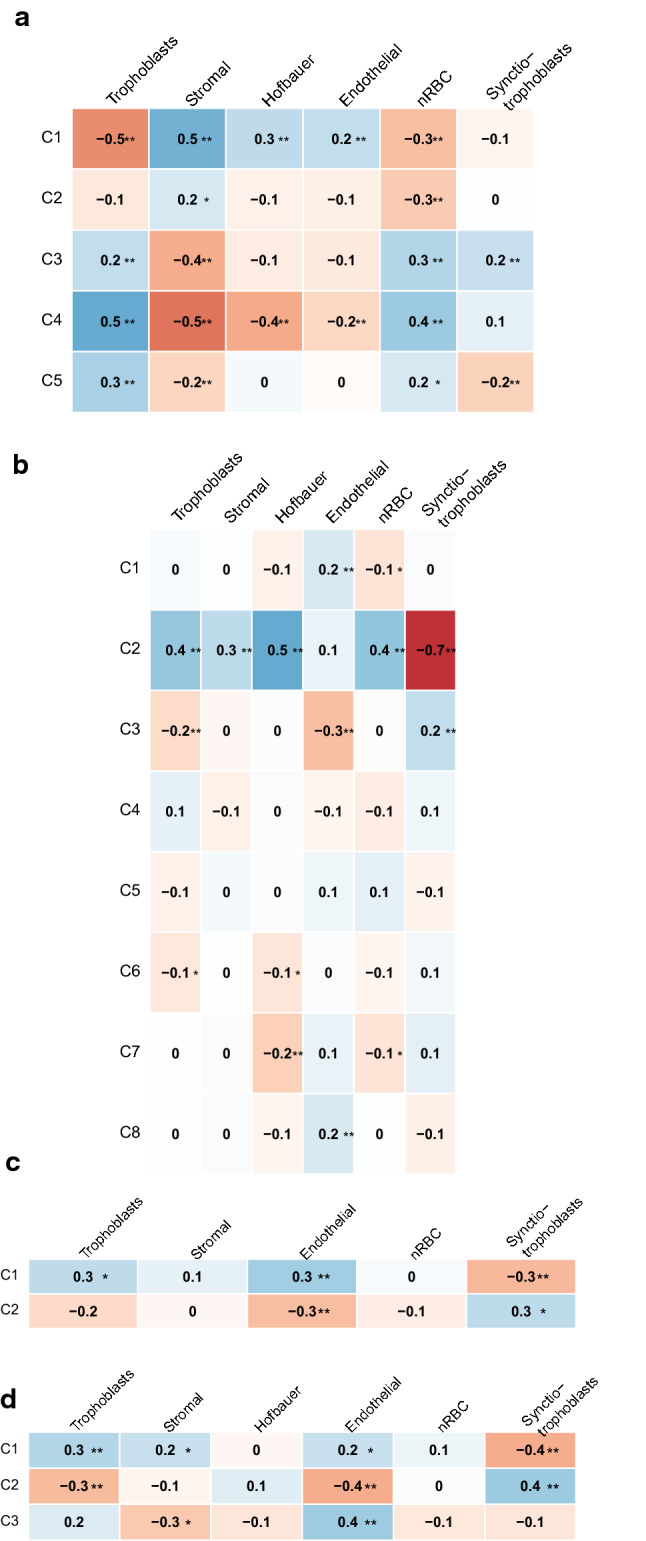
Plot of the Spearman correlation coefficients (**p < 0.001, *p < 0.01) between reference-based and reference free estimated cell types in **a** first trimester placenta (CVS) from ITU, **b** term placenta form ITU, **c** term placenta from PREDO and **d** term placenta from the BET study

**Fig. 2:**
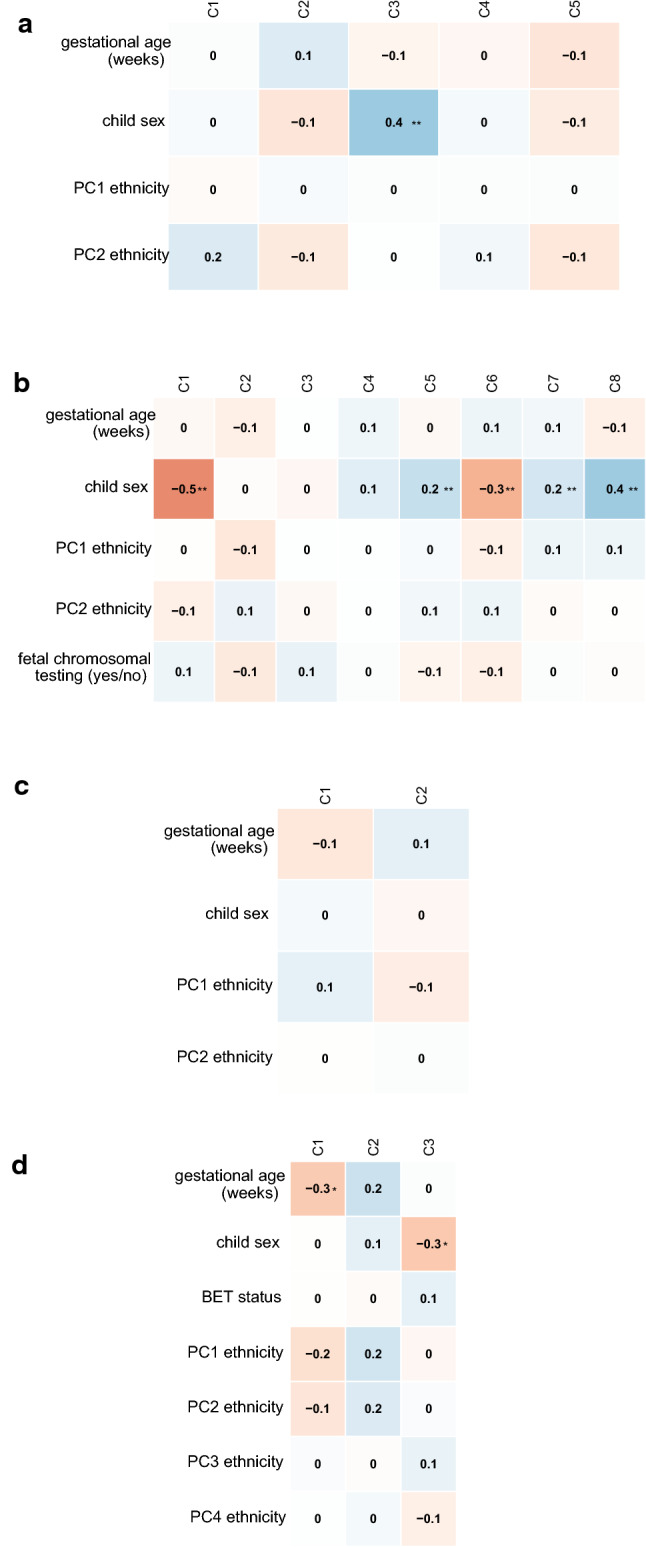
Plot of the Spearman correlation coefficients (**p < 0.001, *p < 0.01) between reference free estimated cell types and phenotypes in **a** first trimester placenta (CVS) from ITU, **b** term placenta form ITU, **c** term placenta from PREDO and **d** term placenta from the BET study

For an illustration of the correspondence between reference-based and reference-free estimated cell types, Spearman correlation coefficients are shown in Fig. 1. Although there were some correlations between reference-based and reference-free estimated cell types, there was no clear matching between reference-based estimated cell types and specific reference-free components. Furthermore, Spearman correlation coefficients for reference-free estimated cell types and included phenotypes are depicted in Fig. 2. It can be seen that especially child sex was correlated with the reference-free estimated cell type components.

### For the majority of data sets, reference-based methods predict variability of DNAm better than reference-free methods

To evaluate the impact of phenotypic variables (GA, child sex, ethnicity) vs. reference-based vs. reference-free cell type composition on the main variance in DNAm (PC1), we compared the predictive performance of six competing models: an intercept-only model (model 1), phenotype model (model 2), reference-based cell type model with or without phenotypes (model 3 and 4) and reference-free cell type model with or without phenotypes (model 5 and 6). All models were tested in each data set among individuals with complete information available (*n* = 200 for CVS from ITU, *n* = 425 for term placenta from ITU, *n* = 118 for term placenta from PREDO and *n* = 136 for term placenta from the BET study with five outliers excluded (see “Materials and methods”) resulting in *n* = 131).

**Fig. 3:**
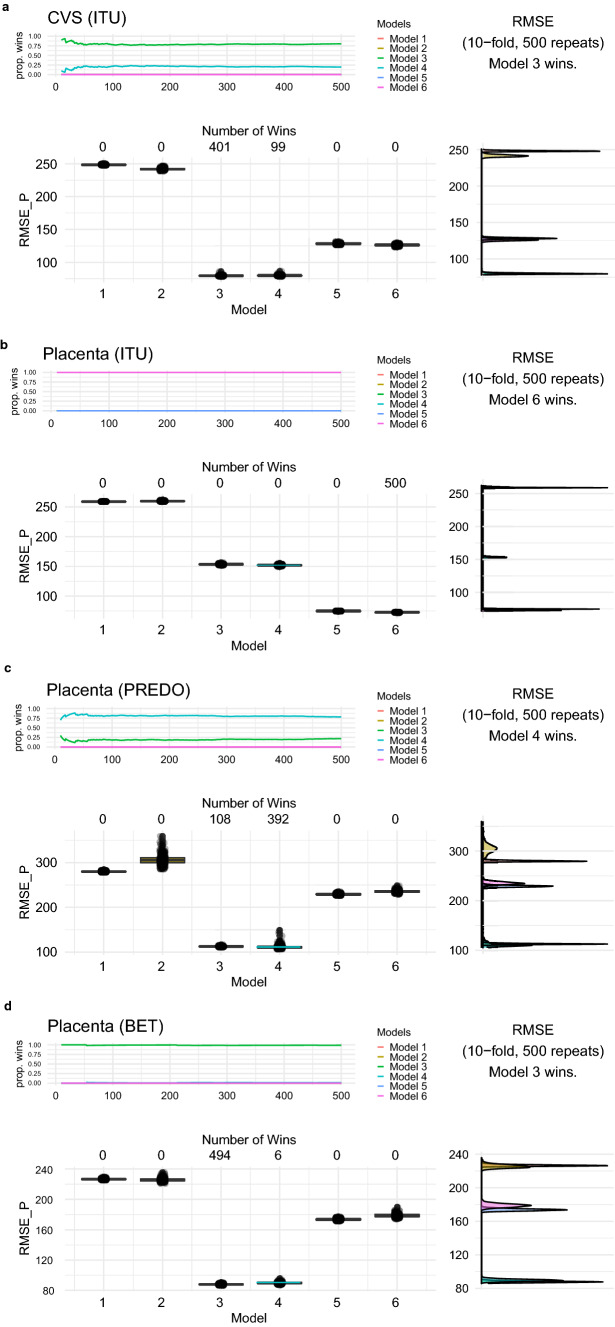
Cross-validation results for predicting PC1 of DNAm comparing 6 models (model 1 = intercept-only; model 2 = phenotypes (gestational age (GA), child sex, ethnicity); model 3 = reference-based estimated cell types; model 4 = reference-based estimated cell types and phenotypes; model 5 = reference-free estimated cell types; model 6 = reference-free estimated cell types and phenotypes). The upper panel illustrates the proportions of wins among all repetitions for each model (models with zero wins overlap and hence not all colors are displayed), and the winning model is listed. The panel below shows the boxplots of the prediction error (root mean square error of prediction, *RMSE*_p_) for all six models with the number of wins for each model displayed at the top. The panel on the right is a graph of density estimates for the prediction errors. Models were compared independently in four different tissue samples, **a** first trimester placenta (CVS) from ITU, **b** term placenta form ITU, **c** term placenta from PREDO and **d** term placenta from the BET study

The results of the cross-validation procedure for model selection are shown in Fig. 3. Models including cell type estimations always performed better than the intercept-only model (model 1) or a model including only phenotypes (GA, sex, ethnicity; model 2). In CVS data (Fig. 3a), the model including reference-based cell types only (model 3) gave the most accurate out-of-sample predictions of PC1 (80% of the wins), with an average prediction error of 79.58 (95% CI [78.57, 80.89]), followed by the model including reference-based cell types and phenotypes. The adjusted *R*^2^ of the winning model was *R*^2^_Adjusted_ = 0.90.

Placental samples taken from the fetal side at birth in the ITU cohort were the only data set where reference-free cell types outperformed reference-based cell types in the prediction of PC1 DNAm (Fig. 3b). In this data set, the model including both reference-free cell types and phenotypes (model 6) always won, presenting with an average prediction error of 72.62 (95% CI [71.97, 73.34]). The adjusted *R*^2^ of the winning model was *R*^2^_Adjusted_ = 0.92. These results did not change when information about fetal chromosomal testing (yes or no) was included as an additional phenotype variable in the models. In PREDO (Fig. 3c), where the placental samples were taken from the decidual side at birth, the model including reference-based cell types together with phenotypes (model 4) performed best (79% of wins) with an average prediction error of 111.44 (95% CI [107.08, 121.70]. In the BET study (Fig. 3d), where placental biopsies spanning from the decidual to the fetal side were collected at birth, the model including reference-based cell types (model 3) won in most of the repeats (99% of wins) with an average prediction error of 87.84 (95% CI [86.48, 89.54]. When including BET (administered or not) as a phenotype variable for the BET study, the winning model was still model was still the model including only reference-based estimated cell types (model 3). The adjusted *R*^2^ of the winning model was *R*^2^_Adjusted_ = 0.86 in both the PREDO and BET placenta.

In both PREDO and BET, the second-best model was the other model including either both reference-based estimated cell types and phenotypes (model 4, for BET) or only reference-based cell types (model 3, for PREDO).

The conclusions from predicting DNAm variability in single CpGs by either reference-based or reference-free estimated cell types were concordant with the model for PC1 in DNAm. On average, reference-based cell types explained more variance (adjusted *R*^2^) in DNAm compared to reference-free cell types among CpGs in CVS from ITU (*n* = 264; *R*^2^_Adjusted_ *M* = 0.13, *SD* = 0.17 vs. *M* = 0.12, *SD* = 0.12), and in placental tissues at birth in PREDO (*n* = 139; *R*^2^_Adjusted_ *M* = 0.11, *SD* = 0.16 vs. *M* = 0.05, *SD* = 0.06), and in BET (*n* = 137; *R*^2^_Adjusted_ *M* = 0.10, *SD* = 0.13 vs. *M* = 0.06, *SD* = 0.07). Only placental tissues sampled at birth in ITU (*n* = 470), reference-free estimated cell types explained more of the variance in DNAm (*R*^2^_Adjusted_ *M* = 0.18, *SD* = 0.18) than reference-based estimated cell types (*R*^2^_Adjusted_ *M* = 0.11, *SD* = 0.15).

### CpGs with larger proportions of variability explained by reference-based cell types map to placenta-specific genes

CpGs where estimated cell type composition explained more than 30% of variance (adjusted *R*^2^ > 0.3) in all four data sets were considered as CpGs at which variability was relatively strongly influenced by cell type proportions. A list of these CpGs and corresponding genes can be found in Supplementary Table S2. For the reference-based model, this was the case for 26,092 CpGs mapping to 8511 genes. For the reference-free model, this was true for 531 CpGs mapping to 398 genes.

**Fig. 4:**
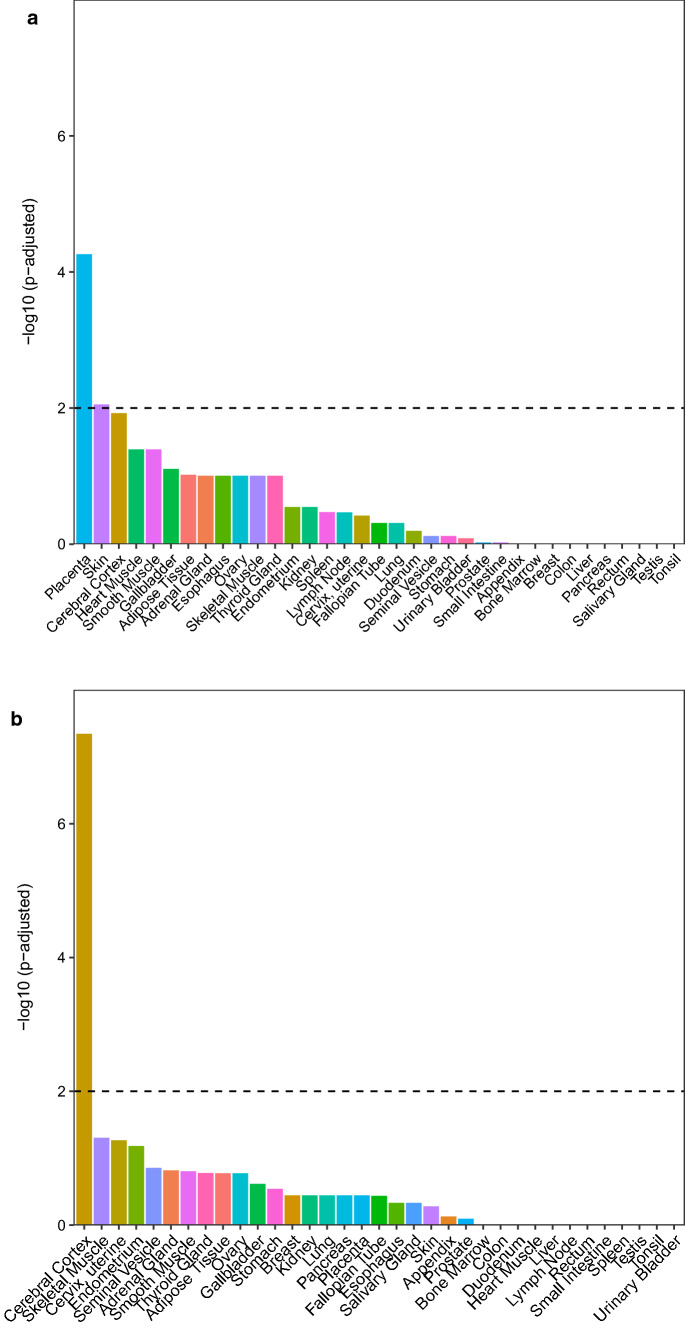
Tissue enrichment among genes mapped to CpGs with a minimum of 30% explained variance in DNAm predicted by cell type proportions from **a** reference-based cell type estimation and **b** reference-free cell type estimation

The results of the tissue enrichment analyses can be seen in Fig. 4. When using the reference-based estimated cell types, genes mapping to CpGs where variability was strongly influenced by cell types were enriched for placenta-specific genes (Fig. 4a, *p* < 0.001 and fold-change = 1.291. We provide a list of these 186 placenta-specific genes in Supplementary Table S3. For reference-free estimated cell types, genes mapping to CpGs where variability is strongly influenced by cell types were not enriched for placenta-specific genes (Fig. 4b): only 10 genes were found to be placenta-specific. However, there was an enrichment for cerebral cortex, with *p* < 0.001, fold-change = 2.209.

**Fig. 5:**
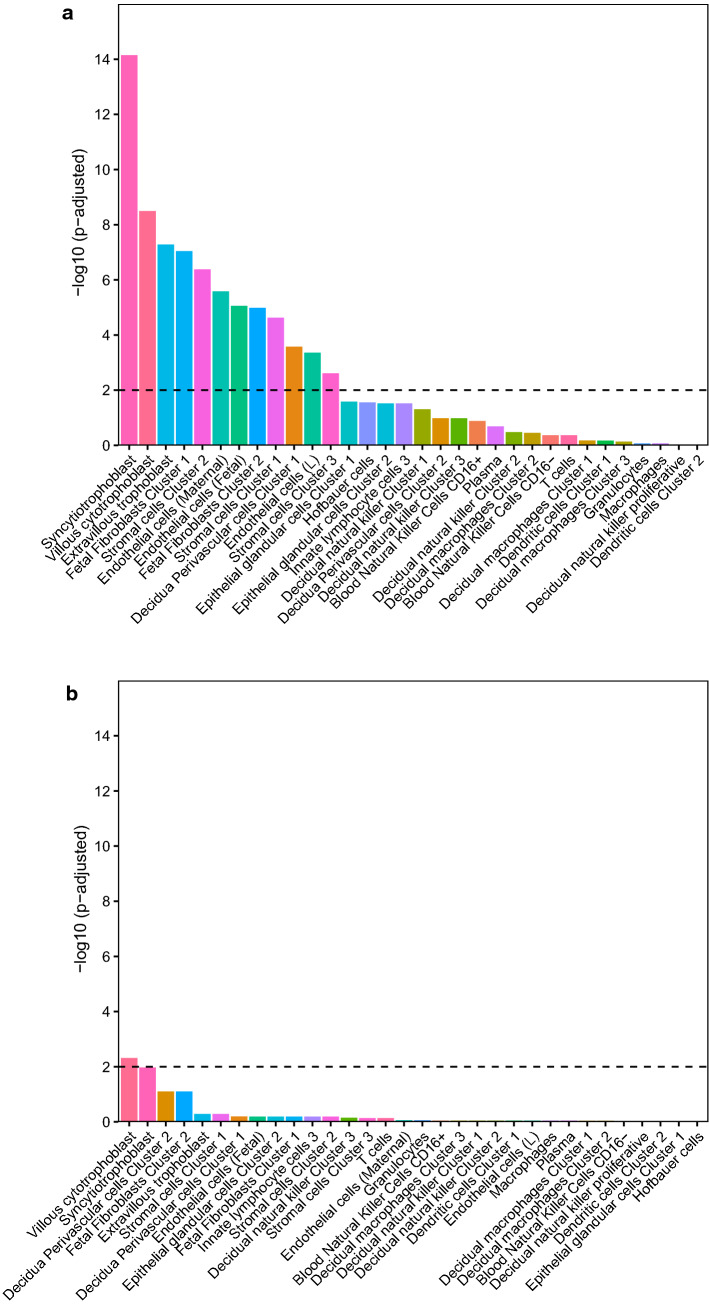
Enrichment for placental cell-specific genes among genes mapped to CpGs with a minimum of 30% explained variance in DNAm predicted by cell type proportions from **a** reference-based cell type estimation and **b** reference-free cell type estimation

Next, we ran cell-specific enrichment analysis using a placenta-specific dataset (*PlacentaCellEnrich* Tool). Cell-specific expression patterns can be seen in Fig. 5. Again, the results reflect a higher placenta-specificity when using the reference-based approach (Fig. 5a), showing a significant enrichment for a number of placental cells as follows: syncytiotrophoblasts, villous cytotrophoblast, extravillous trophoblast, fetal fibroblasts, stromal cells, endothelial cells and decidua perivascular cells. These represent the major cell types in the placenta [41], indicating that this approach accounted for the majority of confounding possible from cell type heterogeneity. Using the reference-free approach (Fig. 5b) there was only an enrichment of villous cytotrophoblasts. A summary of parameters of the cell-specific enrichment can be found in Supplementary Table S4.

### Cell type composition

**Fig. 6:**
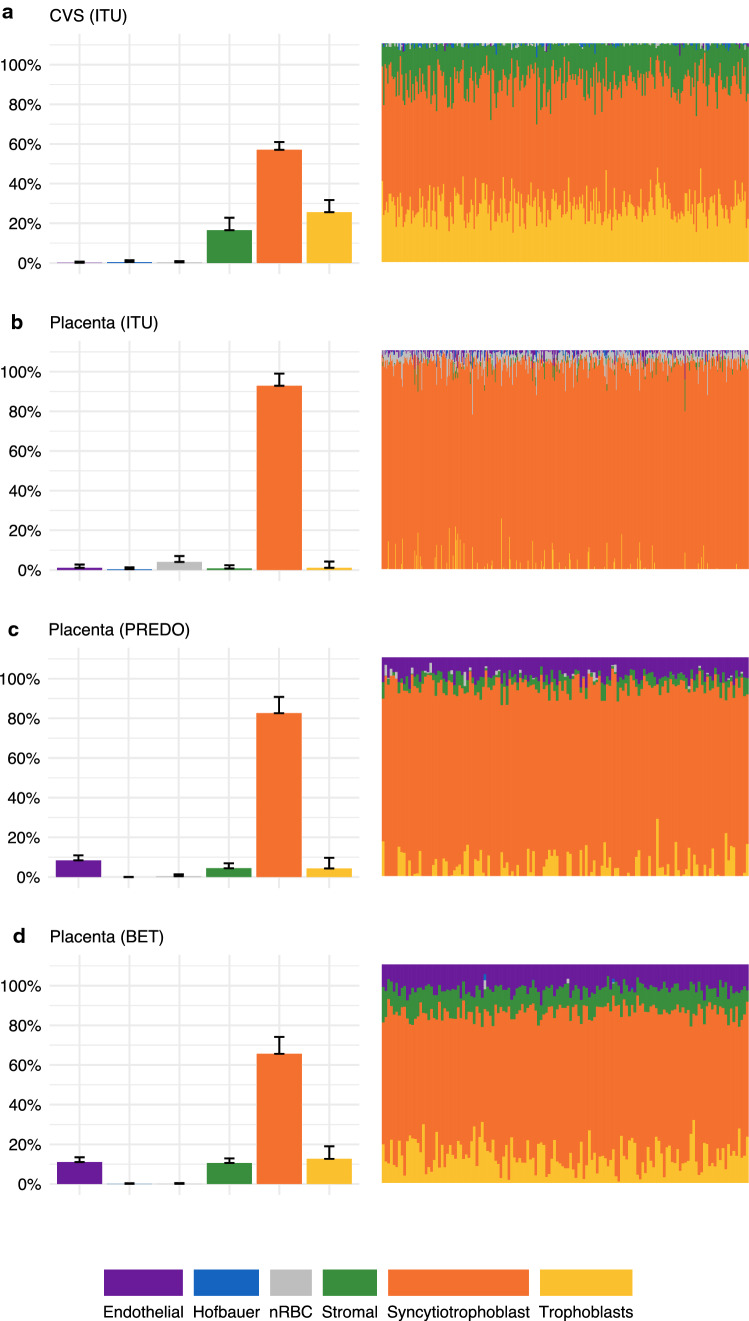
Depicted are the mean and standard deviation of the reference-based estimated cell type’s proportion (raw estimates using the reference by Yuan et al. [17] and robust partial correlation algorithm) together with an illustration of the relative estimated cell type proportion in **a** *n* = 264 individuals in CVS from ITU, **b** *n* = 470 individuals in term placenta from ITU, **c** *n* = 139 individuals in term placenta from PREDO and **d** *n* = 137 individuals in term placenta from the BET study

We next wanted to estimate the cell type proportions in the different study samples using the reference-based method (Fig. 6).

#### Cell type proportions in term placentas show differences between studies

While cell type estimates were highly similar for samples within a study, we observed significantly different estimated cell type proportions among the three studies with placental samples collected at birth, according to each of the four test criteria (ANOVA type, Lawley-Hotelling type, Bartlett–Nanda–Pillai type, and Wilks’ lambda type). Test statistics are given in Supplementary Table S5. Nonparametric relative effects, quantifying the probability that a value obtained from one study sample is larger than a value randomly chosen from the other study samples, are provided in Supplementary Table S6. The post-hoc testing procedure following the global test determined that samples from all three studies and all cell types contributed to these significant differences. In all three term placenta data sets, syncytiotrophoblasts were the main estimated cell type, but the highest proportion was estimated in term placenta from ITU. Estimates for proportions of trophoblasts, stromal and endothelial cells were highest in the BET study sample, followed by term placenta from PREDO.

#### Cell type proportions show intra-individual changes from CVS to term Placenta

The estimated cell type proportions differed significantly between early-pregnancy CVS and placenta sampled at birth for a number of cell types. Largest differences in estimates were observed for stromal cells (*Mdn* = 17.4% in CVS vs. *Mdn* = 0.0% at birth, *Z* = 8.0, *p* < 0.001), syncytiotrophoblasts (*Mdn* = 56.9% in CVS vs. *Mdn* = 95.3% at birth, *Z* = − 8.0, *p* < 0.001), and trophoblasts (*Mdn* = 24.8% in CVS vs. Mdn = 0.0% at birth, *Z* = 8.0, *p* < 0.001) followed by endothelial cells (*Mdn* = 0.0% in CVS vs. *Mdn* = 0.4% at birth, *Z* = − 6.1, *p* < 0.001), nRBC (*Mdn* = 0.0% in CVS vs. *Mdn* = 3.2% at birth, *Z* = − 7.7, *p* < 0.001). This was based on 85 individuals from the ITU cohort for whom both CVS and placenta tissue at birth were available. Syncytiotrophoblasts were the most abundant estimated cell type in both CVS and term placenta tissue, but there was a strong median increase of 38.4% in this cell type from early-pregnancy to birth. The largest decrease from early-pregnancy to birth was in estimated trophoblasts from CVS to term placenta (median decrease of 24.8%), followed by estimated stromal cells (median decrease of 17.4%).

#### Associations between reference-based estimated cell types and gestational age

Finally, we wanted to see whether the estimated cell type proportions follow physiological changes over gestation.

**Fig. 7:**
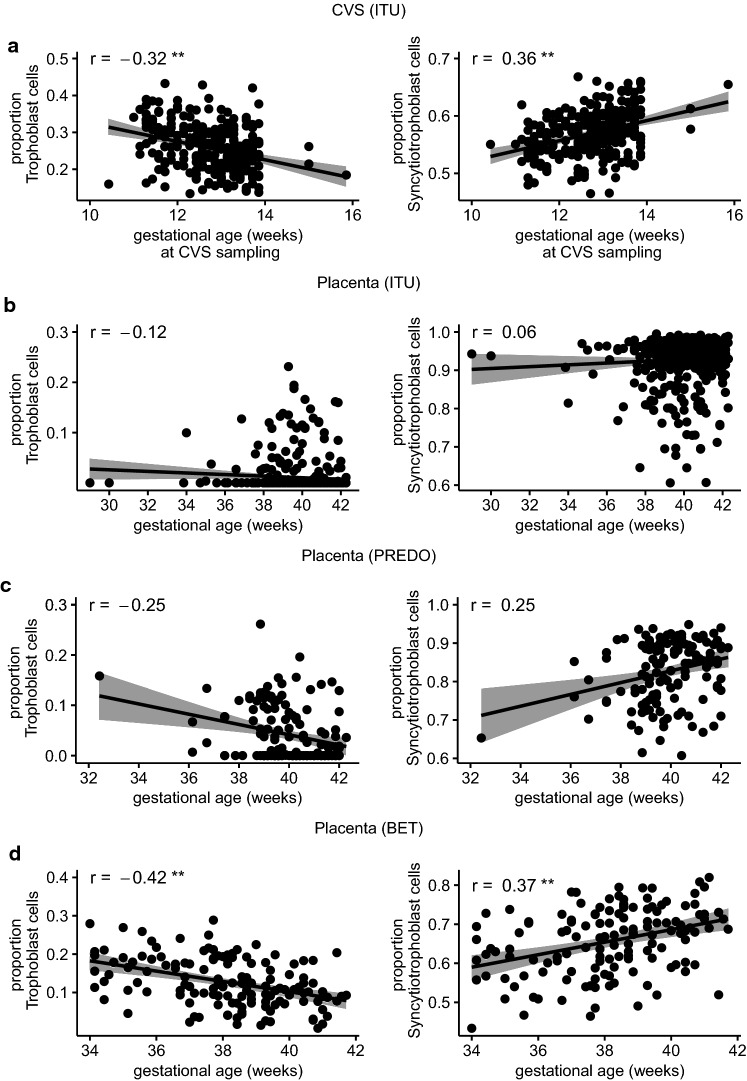
Scatterplots showing the Spearman correlation (**p value < 0.001) of trophoblast and syncytiotrophoblast proportions with gestational age in **a** first trimester placenta (CVS) from ITU (*n* = 264), **b** term placenta form ITU (*n* = 470), **c** term placenta from PREDO (*n* = 139) and **d** term placenta from the BET study (*n* = 137)

Higher GA at sampling was significantly related to lower estimated trophoblast proportions in CVS (*r*_s_ = − 0.32, *p* < 0.001) and term placenta from the BET study (*r*_s_ = − 0.42, *p* < 0.001), and to higher estimated syncytiotrophoblast proportions in CVS (*r*_s_ = 0.36, *p* < 0.001) and term placenta from the BET study (*r*_s_ = 0.37, *p* < 0.001). The effects were not significant, though in the same direction, for the other two data sets (term placenta from ITU and PREDO), where GA was more skewed towards higher gestational age. The relationship of estimated trophoblast and syncytiotrophoblast proportions with GA is shown in Fig. 7.

We observed no significant relationships with GA among the other estimated cell types.

Similar to Yuan et al. [17] we observed no significant sex-specific differences in estimated cell type proportions in any of the study samples.

## Discussion

In this study, we examined a new DNAm-based reference which enables reference-based cell type estimation in placenta [17] in a large data set comprising over 1000 samples from three independent studies, with *n* = 746 placental samples collected at birth, and *n* = 264 during the first trimester of pregnancy. We investigated intra- as well as inter-individual differences in estimated cell type proportions. Furthermore, we compared the reference-based to a reference-free approach (namely, RefFreeEWAS) [31], regarding its potential to control for cell type proportions in DNAm studies of human placenta. We provide lists of CpGs from the EPIC array which we found to be (1) non-variable in placental tissue (Supplementary Table S1), and (2) highly influenced by cell types (Supplementary Table S2).

Using a cross-validation model focusing on the prediction of the major variance in DNAm, as well as an investigation at single CpGs level, we confirmed the importance of cell type composition for variability in DNAm.

At the same time, the latter shows that it is a select subset of CpGs where the impact of cell type proportions on DNAm is especially important (Supplementary Table S2).

Both reference-free and reference-based cell type estimation methods can account for variability in DNAm. However, for the majority of data sets, the reference-based approach better predicted variability of DNAm.

Generally, reference-based cell type estimation allows for a more direct interpretation of cell type composition. This was underscored by the fact that the overlap in CpGs with high amount of DNAm variability explained by estimated placental cell types was much more consistent among the different data sets when using reference-based cell types (26,092 CpGs) versus reference-free cell types (531 CpGs). Furthermore, genes mapping to these CpGs with high proportions of DNAm variability explained by estimated reference-based cell types were enriched for placenta-specific genes, while this was not the case when using the reference-free approach (see Fig. 4). A possible reason for this could be that the reference-free methods do not only depict cell types, but further unknown sources of variance, and as such it is difficult to interpret what the estimated reference-free ‘cell types’ actually reflect. This also becomes clear from Figs. 1 and 2, where we depict that reference-based estimated cell types are not highly correlated with a specific reference-free cell type component, but rather with child sex. This might also explain why in one of the term placenta data sets DNAm variability was better explained by reference-free compared to reference-based estimated cell types - probably not only cell types were covered by the estimated ‘cell types’ which contributed to DNAm variability in the complex tissue samples. This could suggest that even though reference-based cell type correction approaches outperform reference-free approaches in most settings, cohort-specific differences may affect the performance of these methods.

Overall, considering the performance of the reference-based cell type estimation, it may be advisable to use reference-based methods, such as from Yuan et al. [17] in future studies investigating DNAm in human placenta.

Higher GA was associated with higher proportions of syncytiotrophoblasts and lower proportions of trophoblasts in the placenta samples collected at birth (Fig. 7). This finding was congruent with the changes in estimated cell type composition we observed from first trimester to birth placenta samples from the same individuals: trophoblast cells showed the largest decrease, syncytiotrophoblasts the largest increase. These differences in the estimated cell type proportions between early and late pregnancy are probably reflective of placental maturation process [42]. Trophoblasts give rise to further subpopulation of cells and syncytiotrophoblasts expand during pregnancy [5]. Yuan et al. [17] reported an increase in estimated syncytiotrophoblasts and endothelial cells and decrease in stromal cells from first trimester to term placenta samples, which is again concordant with our results despite their comparison of samples from different individuals, in contrast to our within-sample design in 85 individuals. Nevertheless, it should be mentioned that we cannot rule out that some of the differences in estimated cell type proportions may arise from differences in sampling and storage conditions of the CVS and the placental tissue.

Regarding child sex, Yuan et al. did not find any association with estimated cell composition [17]. We can confirm this result, as there was no evidence for sex-specific differences in reference-based estimated cell type composition.

Additionally, the use of three independent studies (ITU, PREDO, BET) enabled us to investigate between-study differences in estimated cell type proportions at birth. We observed that cell type composition was rather consistent among samples within a study but different between studies. The larger variance in cell type proportions between studies (versus between individuals within a study) might reflect the different sampling schemes of placental tissue (see “Materials and methods”). The placenta is a highly complex organ, which makes the sampling procedure difficult and particularly prone to differences between studies [21, 43].

An important strength of our study is that we were able to investigate placental cell type composition in a large number of placentas from different independent studies. In addition to examining placental DNAm at birth, we included early-pregnancy placental CVS samples: in a subset of 85 individuals, longitudinal data on placental DNAm both in early pregnancy and at birth were available, giving us the rare chance to examine change over time within the same placentas. We also provide resources that can be used for the interpretation and design of DNAm studies in placenta, especially EWAS. However, there are also some limitations: we rely on bioinformatic indirect deconvolution, which also limits our investigation to the cell types included in the reference sample [17]. This was in turn limited by the availability of unique markers suitable for cell type selection using fluorescence-activated cell sorting, and dissection accuracy. Future tools based on single-nucleus DNA methylation analyses would undoubtedly improve cell type accuracy as well as diversity, thus improving usefulness for deconvolution in bulk tissue analyses. Furthermore, we only compared one reference-based deconvolution to one of several (semi-) reference-free approaches available [16]. Thus, our comparison of performance between methods is limited to these chosen approaches and is only an indication of the ability of the reference-based method to account for variability in DNAm compared to another often-used reference-free approach, but not generalizable to all reference-free methods. Additionally, we only used the first principal component of DNAm in the cross-validation procedure for model comparison, which is a reduction of dimensionality and improves interpretability, but at the same time can only capture part of the total variation in the data.

Overall, addressing cell type heterogeneity in studies of DNAm is important to avoid misinterpretation of results, to limit confounding and increase precision by distinguishing changes in cell type proportions from epigenetic changes due to other factors, such as for example environmental exposures [44]. Apart from this, cell type composition is also an important factor to consider for understanding gene regulatory mechanisms in human tissues [45] and tissue function overall. This study contributes to a more detailed understanding of the interrelation between DNAm and estimated cell type composition in human placenta and stands as a resource to help researchers design future DNAm studies of human placenta and interpret results of both existing and future studies.

## Supplementary Information

Below is the link to the electronic supplementary material.

Scree plot of the principal component analysis of DNAm beta values in a) CVS from ITU (*n* = 264), b) placenta from ITU (*n* = 470), c) placenta from PREDO (*n* = 139) and d) placenta from the BET study (*n* = 137) (PDF 22 KB)

Illustration of samples identified to be different (n = 16) in fetal term placenta from the ITU cohort. Shown is the a) Boxplot of PC1 of DNAm with the outlier samples (greater than three times inter-quartile-range) colored in red, b) Average sample-sample correlation (Spearman's correlation) among DNAm beta values for each sample, with the previously identified outliers colored in red, and c) Cell type proportions of reference-based estimated cell types in term placenta form ITU with the respective samples in red (PDF 64 KB)

Impact of samples presenting with different estimated proportions of Hofbauer and nRBC cells (n = 5) in the BET study sample with complete information (n = 136) on the cross-validation model. Depicted are a) Cross-validation results for predicting PC1 of DNAm comparing 6 models (model 1 = intercept-only; model 2 = phenotypes (gestational age, child sex, ethnicity); model 3 = reference-based estimated cell types; model 4 = reference-based estimated cell types and phenotypes; model 5 = reference-free estimated cell types; model 6 = reference-free estimated cell types and phenotypes) in the BET study sample, showing the boxplots of the prediction error (root mean square error of prediction, *RMSE*p) for all six models with the number of wins for each model displayed at the top; b) Scatterplot of reference-based estimated cell type proportions against PC1 of methylation beta values; c) Boxplot of reference-based estimated cell type proportions. In Figure S3b it can be seen that all samples in the BET study apart from five (of which two overlap between nRBC and Hofbauer) have no estimated proportion of Hofbauer and nRBC cells. These five samples lead to an instability in the cross-validation model shown in Fig. S3a (PDF 210 KB)

Histogram of adjusted *R*^2^ values for all CpGs resulting from linearly regressing DNAm beta values on reference-based versus reference-free estimated cell types in a) CVS form ITU, b) placenta from ITU, c) placenta from PREDO and d) placenta from the BET study (PDF 9 KB)

CpGs identified as non-variable defined as having less than 5% range in DNAm beta values between the 10th and 90th percentile (RefRange) (XLSX 3632 KB)

CpGs with a minimum of 30% explained variance (*R*^2^_Adjusted_) in DNAm predicted by cell type proportions from reference-based cell type estimation and reference-free cell type estimation with their corresponding genes (XLSX 613 KB)

List of 186 placenta-specific genes belonging to CpGs with a minimum of 30% explained variance (*R*^2^_Adjusted_) in DNAm predicted by cell type proportions from reference-based cell type estimation, identified by tissue enrichment analysis. Genes are further grouped as tissue enriched (genes with an expression level greater than 1 (TPM or FPKM) that also have at least five-fold higher expression levels in placenta compared to all other tissues), group enriched (genes with an expression level greater than 1 (TPM or FPKM) that also have at least five-fold higher expression levels in a group of 2-7 tissues compared to all other tissues, and that are not considered tissue enriched) and tissue enhanced (genes with an expression level greater than 1 (TPM or FPKM) that also have at least five-fold higher expression levels in placenta compared to the average levels in all other tissues, and that are not considered tissue enriched or group enriched) (XLSX 12 KB)

Results of cell-specific gene enrichment among genes mapped to CpGs with a minimum of 30% explained variance in DNAm predicted by cell type proportions from a) reference-based cell type estimation and b) reference-free cell type estimation (XLSX 13 KB)

Nonparametric test statistics for the comparison of cell type proportions between three term placenta data sets. Empirical nonparametric relative effects for each cell type (XLSX 10 KB)

## Data Availability

Due to the sensitive nature of the patient data used in the current study and consent, the data sets are not and cannot be made publicly available for data protection reasons.

## Code availability

R Code for the main analyses is provided here: https://doi.org/10.5281/zenodo.5713621.
