# Supplementary figures and images for: Reliability of a novel approach for reference-based cell type estimation in human placental DNA methylation studies
Source: Cell Mol Life Sci. 2022 Feb 3;79(2):115. doi: 10.1007/s00018-021-04091-3 (PMC8813756; doi:10.1007/s00018-021-04091-3)

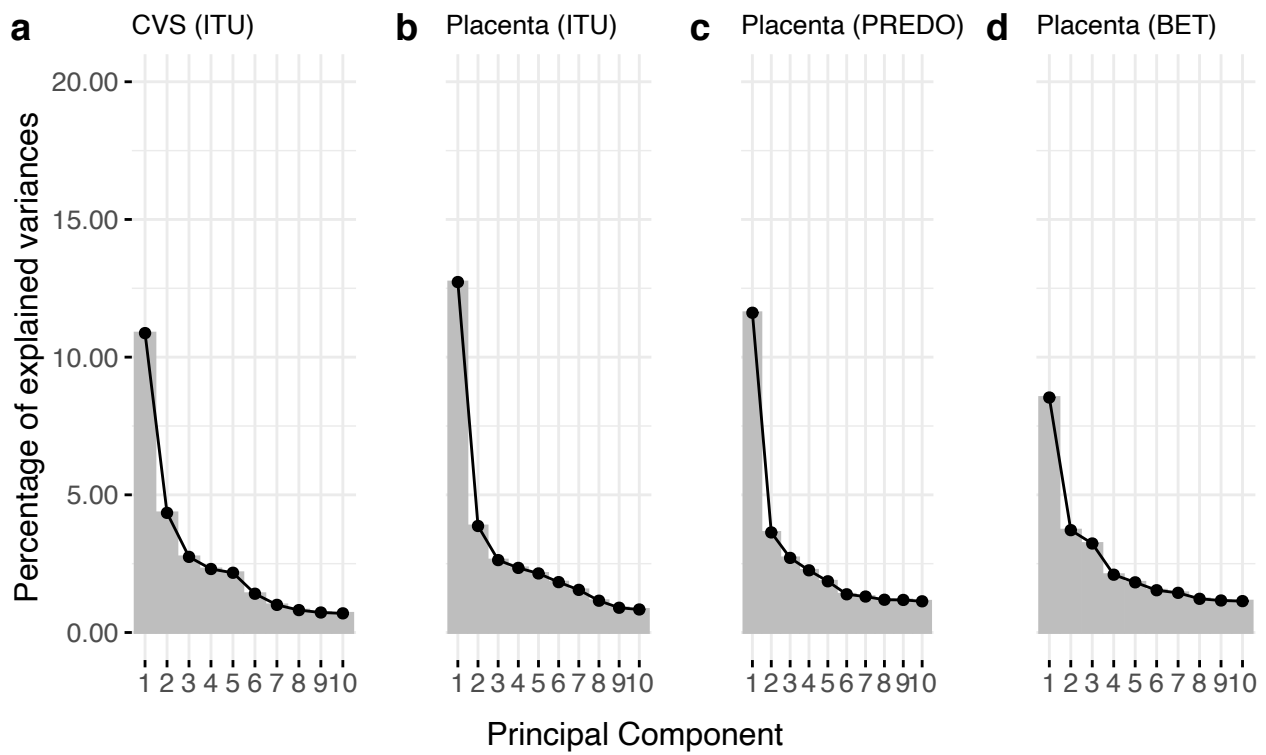

Supplement: Supplementary file 1 — Scree plot of the principal component analysis of DNAm beta values in a) CVS from ITU (n = 264), b) placenta from ITU (n = 470), c) placenta from PREDO (n = 139) and d) placenta from the BET study (n = 137) (PDF 22 KB) [file 18_2021_4091_MOESM1_ESM.pdf]

**a**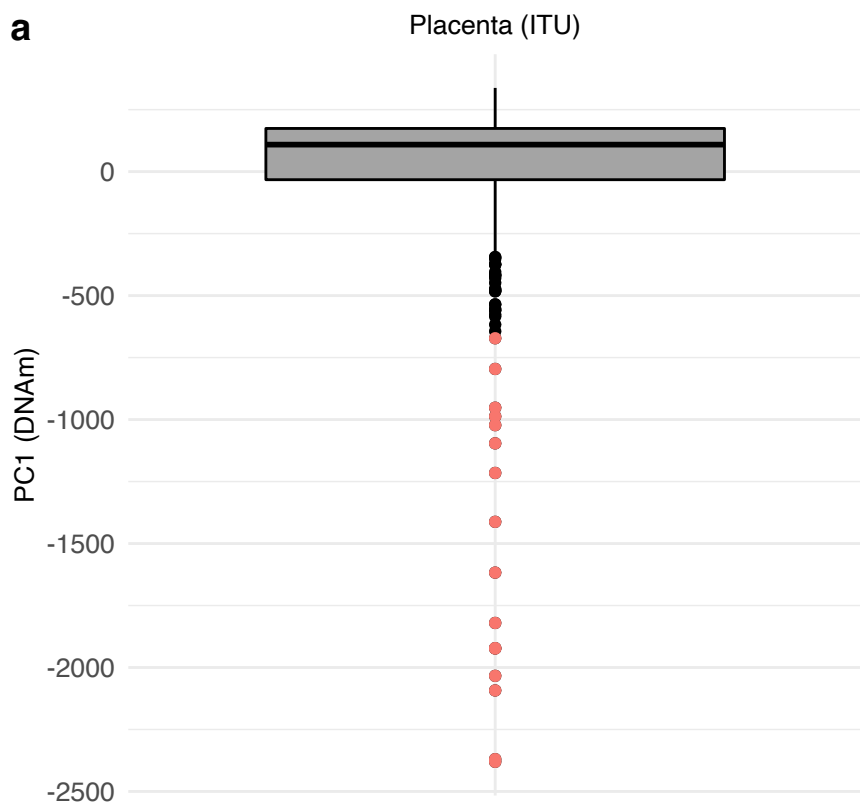**b**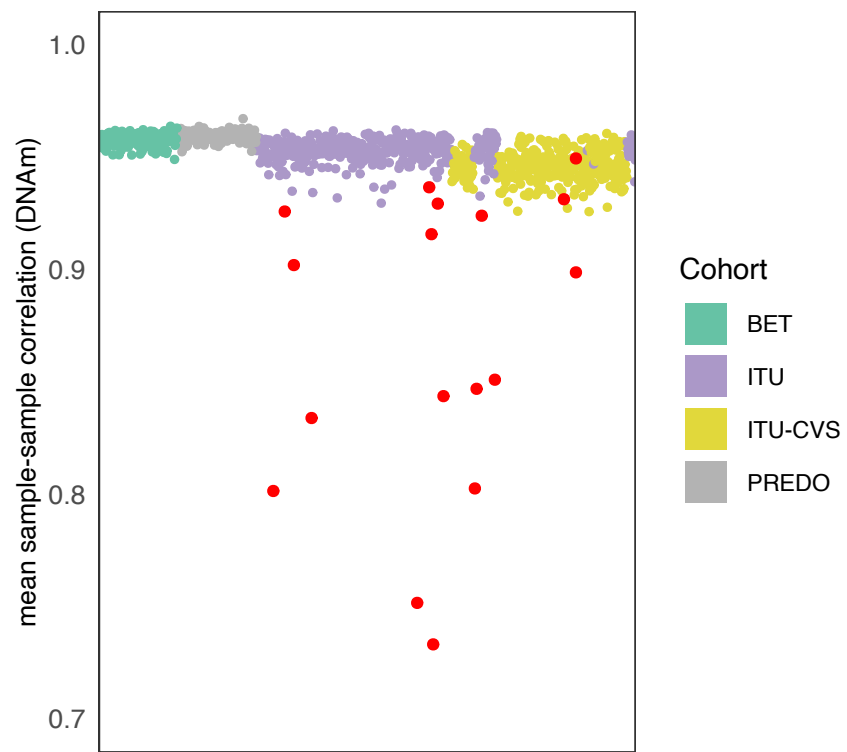**c**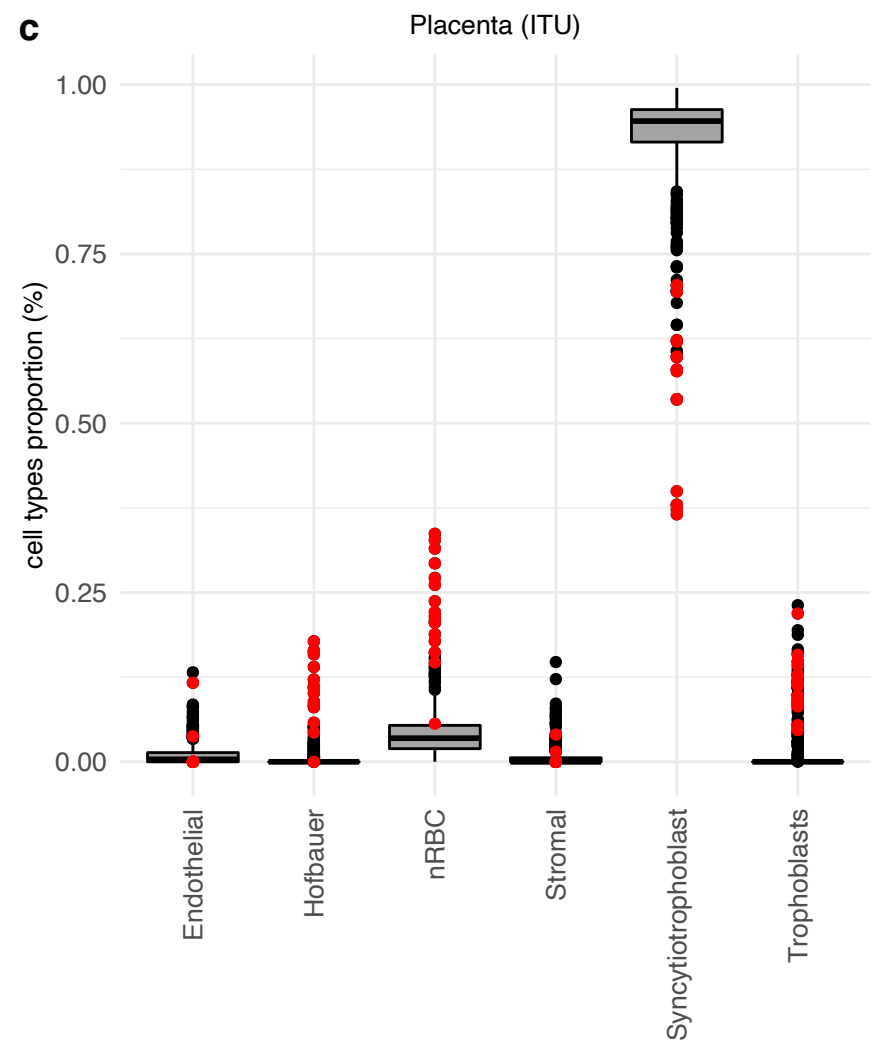

Supplement: Supplementary file 2 — Illustration of samples identified to be different (n = 16) in fetal term placenta from the ITU cohort. Shown is the a) Boxplot of PC1 of DNAm with the outlier samples (greater than three times inter-quartile-range) colored in red, b) Average sample-sample correlation (Spearman's correlation) among DNAm beta values for each sample, with the previously identified outliers colored in red, and c) Cell type proportions of reference-based estimated cell types in term placenta form ITU with the respective samples in red (PDF 64 KB) [file 18_2021_4091_MOESM2_ESM.pdf]

**a**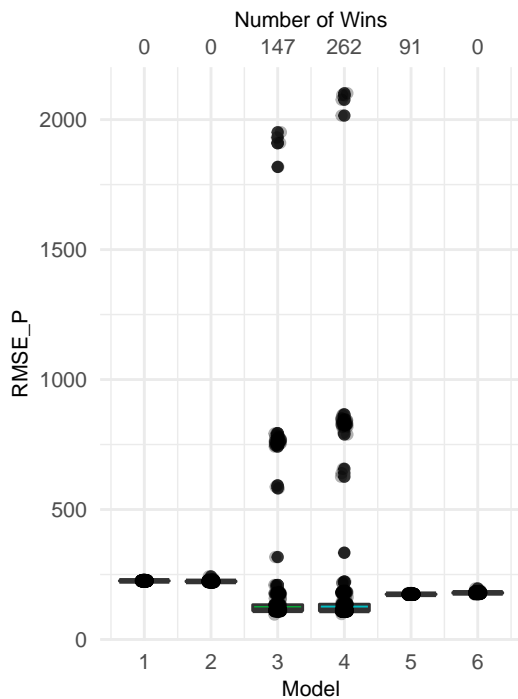**b**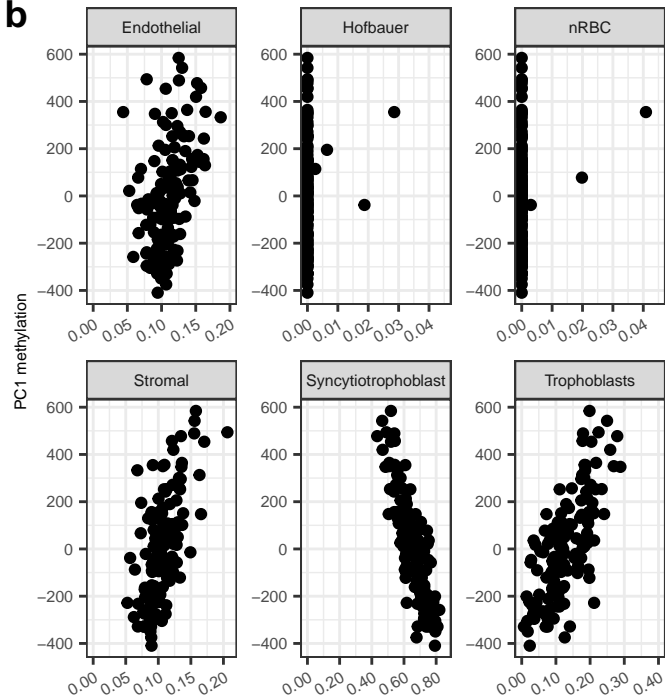**c**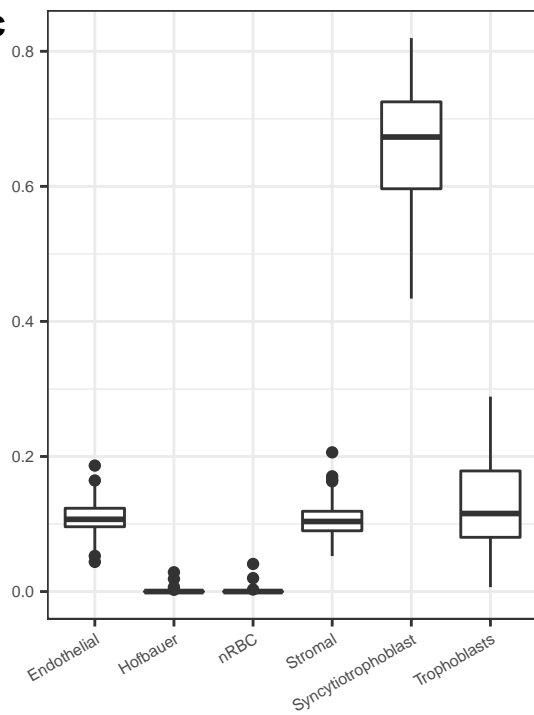

Supplement: Supplementary file 3 — Impact of samples presenting with different estimated proportions of Hofbauer and nRBC cells (n = 5) in the BET study sample with complete information (n = 136) on the cross-validation model. Depicted are a) Cross-validation results for predicting PC1 of DNAm comparing 6 models (model 1 = intercept-only; model 2 = phenotypes (gestational age, child sex, ethnicity); model 3 = reference-based estimated cell types; model 4 = reference-based estimated cell types and phenotypes; model 5 = reference-free estimated cell types; model 6 = reference-free estimated cell types and phenotypes) in the BET study sample, showing the boxplots of the prediction error (root mean square error of prediction, RMSEp) for all six models with the number of wins for each model displayed at the top; b) Scatterplot of reference-based estimated cell type proportions against PC1 of methylation beta values; c) Boxplot of reference-based estimated cell type proportions. In Figure S3b it can be seen that all samples in the BET study apart from five (of which two overlap between nRBC and Hofbauer) have no estimated proportion of Hofbauer and nRBC cells. These five samples lead to an instability in the cross-validation model shown in Fig. S3a (PDF 210 KB) [file 18_2021_4091_MOESM3_ESM.pdf]

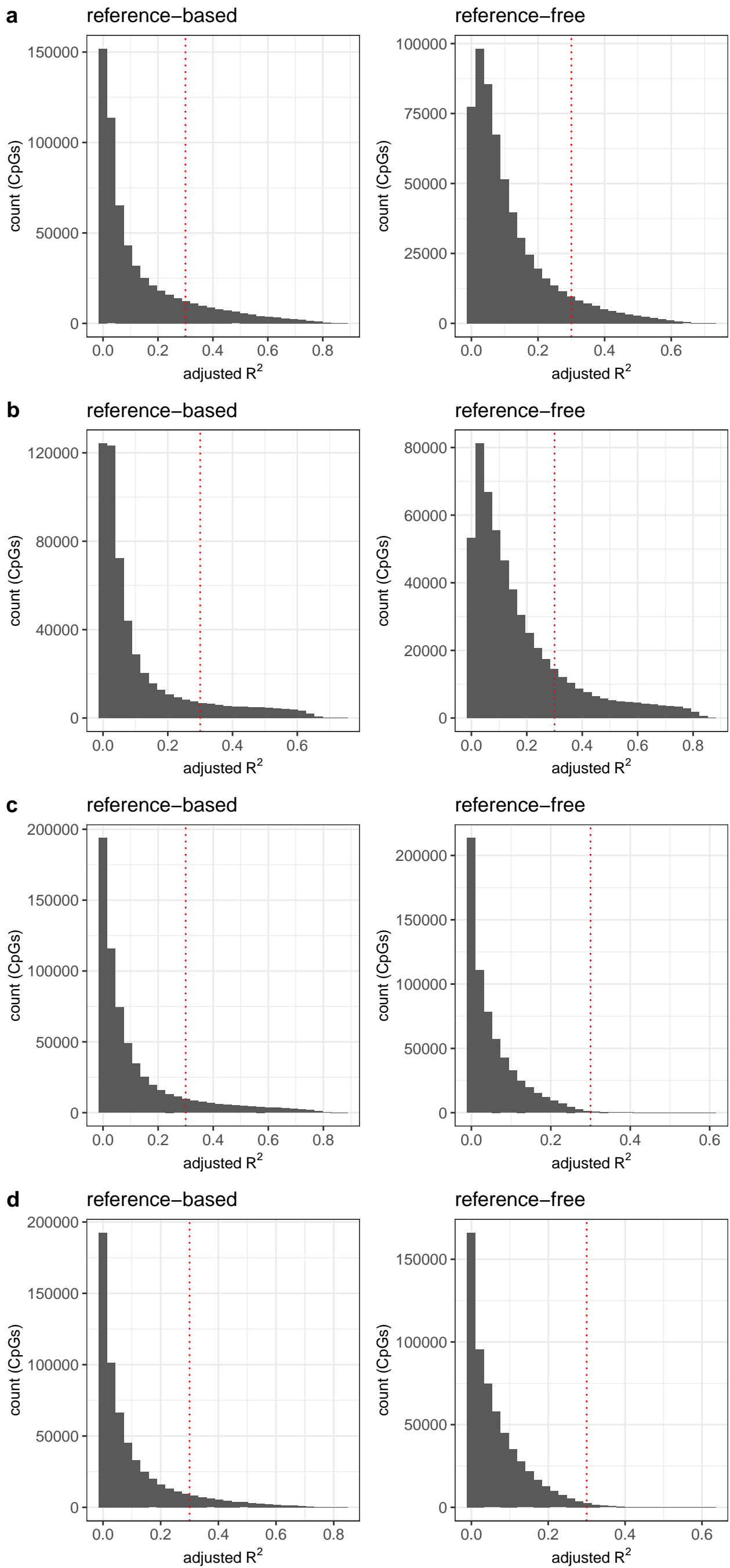

Supplement: Supplementary file 4 — Histogram of adjusted R2 values for all CpGs resulting from linearly regressing DNAm beta values on reference-based versus reference-free estimated cell types in a) CVS form ITU, b) placenta from ITU, c) placenta from PREDO and d) placenta from the BET study (PDF 9 KB) [file 18_2021_4091_MOESM4_ESM.pdf]
